# The use of platelet-rich plasma therapy in treating tennis elbow: A critical review of randomised control trials

**DOI:** 10.1016/j.jcot.2022.101965

**Published:** 2022-07-31

**Authors:** Joshua Rui Yen Wong, Esme Toth, Kannan Rajesparan, Abbas Rashid

**Affiliations:** a University College London, Gower St, London, WC1E 6BT, United Kingdom; b Department of Trauma and Orthopaedic Surgery, University College London Hospitals NHS Foundation Trust, 235 Euston Rd, London, NW1 2BU, United Kingdom

**Keywords:** Platelet-rich-plasma, Steroid injections, Tennis elbow, Systematic review, TE, Tennis elbow, PRP, Platelet-rich plasma, AWB, Autologous Whole Blood, RCTs, Randomised Control Trials

## Abstract

Tennis elbow (TE) is a painful and debilitating condition of the elbow. Recently, the use of orthobiologics, such as platelet-rich-plasma (PRP), has been proposed to promote tendon regeneration. Despite their popularity, there is a paucity of updated reviews on the use of PRP compared with other treatment modalities for treating TE. The aim of this review is to summarise high quality studies that compare the use of PRP therapy with other therapies for TE and to identify areas where further research is warranted.

This systematic review was performed in accordance to the Preferred Reporting Items for Systematic Reviews and Meta-Analysis (PRISMA) guidelines. A comprehensive literature search of PubMed, Scopus and Cochrane Library databases was undertaken in May 2021. Articles were screened for the following criteria: randomised control trials (RCTs) involving PRP in at least one of the treatment arms for tennis elbow. The quality of the RCTs included were analysed for their risk of bias using the modified Cochrane Collaboration Risk of Bias Assessment Tool for randomised trials.

A total of 20 RCTs of which 1520 TE patients were analysed. The RCTs included in this review compared PRP with various treatment modalities routinely used in clinical practice such as physiotherapy, steroid injections, Autologous Whole Blood (AWB) and surgical interventions. With regards to the quality of RCTs, collectively, selection bias was found to be low risk however, performance bias in terms of blinding of participants and personnel performed poorly. Of the 20 RCTs, only 5 studies were classified as low risk of bias. In these 5 studies, 2 RCTs compared PRP with steroids and reported contrasting results, 1 RCT compared PRP with AWB injections which reported both to be similarly efficacious, 3 RCTs included a placebo group and only 1 reported superior effects with PRP. There are 2 main types of PRP classified according to the number of pro-inflammatory leukocyte i.e. leukocyte-rich and leukocyte-poor PRP. However, only 8 studies documented the formulation of PRP used. While the heterogeneity of PRP formulations could in-part explain the reported differences in outcomes, overall there is limited robust evidence to recommend PRP therapy for TE. Further research is required to establish the optimal formulation and administration of PRP injections. Proper documentation of TE patients need to be standardised before concrete recommendations on the use of PRP therapy may be offered.

## 1 Introduction

Tennis elbow (TE), or lateral epicondylitis, is a painful and debilitating condition of the elbow caused by angiofibroblastic hyperplasia of the tendinous origin of wrist extensors especially the extensor carpi radialis brevis (ECRB) muscle.^1^ TE is a common elbow pathology affecting 4–7 people per 1000 annually and is particularly common amongst racquet sports athletes due to overuse or repetitive stress.^2^^,^^3^ TE typically presents as pain over the lateral epicondyle extending distally over the mobile wad that may be exacerbated by forearm supination and radial deviation. TE was previously thought to be a self-limiting condition,^4^ however chronic cases of TE have been reported to persist for years and associated to complications such as muscle wasting and weak grip strength.^5^

In the first instance, the current national treatment guidelines in England proposes a 6-week long plan of analgesia and rest with a reduction or cessation in the aggravating activities. During this period, the application of an orthosis such as a wrist or elbow brace may also be considered. After 6 weeks, other forms of conservative management such as physiotherapy with eccentric loading, strengthening exercises and massage may be considered.^6^^,^^7^ Finally, if the patient shows no improvement after 6–12 months of treatment, referral to an orthopaedic clinic should then be considered.^9^ Surgical debridement of tendinosis including the release or repair of the damaged extensor tendon may then be considered as a last resort for these patients with refractory pain or severe functional impairment. Percutaneous needle tenotomy and injections of corticosteroid into the tendon sheath, platelet-rich plasma (PRP) or autologous whole blood (AWB) are also alternative therapeutic options before resorting to surgical decompression, debridement with or without tendon repair.^3^^,^^6^, ^7^, ^8^, ^9^

Lately, in vitro studies have proposed that PRP can stimulate tendon regeneration as high concentrations of growth factors and cytokines have been found at the site of tendon healing, suggesting a potential mechanism of action.^10^^,^^11^ Additionally, multiple studies demonstrated PRP to be able to positively influence angiogenic factors as well as tendon cell proliferation.^12^^,^^13^ Despite their popularity, there is a paucity of recent and updated reviews on the use of PRP comparing with other available treatment modalities for treating TE. Given that surgical intervention for the treatment of TE is usually only offered after a trial of conservative and medical injection therapy, most TE patients would have undergone a form of injection therapy. Given their importance, it is crucial to scrutinise these injection therapies.

This study aims to summarise the findings of robust studies with high levels of evidence which compare PRP therapy with other available treatment modalities for TE. Based on the current basic science evidence, we hypothesise that PRP therapy is therapeutically efficacious in managing pain and functionality in TE patients.

## 2 Methods

This systematic review was performed in accordance to the Preferred Reporting Items for Systematic Reviews and Meta-Analysis (PRISMA) guidelines. In May 2021, the Cochrane Library, PubMed and Scopus databases were thoroughly searched with the following search string: (“elbow tendon injury” OR “elbow tendinopathy” OR “elbow tendonitis” OR “tennis elbow” OR “lateral epicondylitis” OR “lateral epicondylosis” OR “lateral epicondylalgia” OR “epicondylitis”) AND (“platelet-rich plasma” OR “autologous whole blood”). Each keyword is connected via a Boolean operator “AND” or “OR”.

Articles were screened for the following criteria: randomised control trials (RCTs) involving PRP in at least one of the treatment arms for TE as well as a treatment group without PRP therapy. Articles not in English language, incomplete data, meeting summaries, case reports, retrospective studies, randomised and non-randomised prospective cohort studies, or review articles with no original data were excluded.

Details of each RCT were retrieved individually. Information on the different treatment groups, number of patients included, duration of symptoms, length of follow up and blinding, key outcomes for each RCT are documented in Table 1. The details of the treatment recorded include the type of PRP used and how the PRP was prepared where available. All quantitative and qualitative outcomes, including pain and performance scores, of the RCTs were recorded where available. The primary outcomes compared was improvements in pain and function. Secondary outcomes included any complications or side effects of the treatments. Attempts to contact the corresponding authors of studies which required clarification of certain details were made. The outcomes of the RCTs were largely evaluated qualitatively due to inadequate quantitative data for the collective pooling of the data quantitatively. Additionally, a meta-analysis was not attempted as multiple studies were at risk of bias.

**Table 1:** Summary of RCTs and treatment groups.

Author	Year	Group/Treatment	PRP preparation machine	Administration of injection	No. of patients	Duration of symptoms	Length of follow up	Overall bias risk∗∗	Results	Conclusion
Mishra et al.	2014	1. PRP (leukocyte-enriched) injection group	GPS, Biomet Biologics	Manual Palpation	116	At least 3 months	12 weeks and 24 weeks	Low risk	VAS (% improvement): 71.5∗ PRTEE: 16.17 At 12 weeks (n = 192), the PRP group reported an improvement of 55.1% in their pain scores. At 24 weeks (n = 119), the PRP group reported an improvement of 71.5% in their pain scores. Success rates for the PRP group at 12 and 24 weeks were 75.2% and 83.9% respectively.	At the end of 12 weeks, there no significant difference between PRP and dry needling was found. However, at 24 weeks, clinically meaningful improvements were found in the PRP group.
		2. Active control group (dry needling)	NR	Manual Palpation	114	At least 3 months	12 weeks and 24 weeks		VAS (% improvement): 56.1 PRTEE: 21.06 At 12 weeks, an improvement of 47.4% was seen in the active control group. At 24 weeks, an improvement of 56.1% was seen in the active control group Success rates for control group at 12 and 24 weeks were 65.9% and 68.3% respectively.	
Raeissadat et al.	2014	1. PRP (leukocyte-enriched)	PRP processing: Rooyagen kit PRP Quantification and qualification: Sysmex KX 21	Manual Palpation	31	At least 3 months	1,2,6 and 12 months	High risk	At 12 months: VAS: 3.29 ± 2.41 PPT: 26.9 ± 6.3 MEPS: 78.18 ± 18 Success rates at 12 months follow-up was 75%	There was no statistically significant difference between the PRP and AWB groups in terms of pain scores and success rate in all follow up examinations including 4,8 weeks and 6 and 12 months after initiating therapy.
		2. AWB		Manual Palpation	30	At least 3 months	1,2,6 and 12 months		At 12 months: VAS: 3.94 ± 2.42 PPT: 22.5 ± 5.7 MEPS: 73.16 ± 18 Success rates at 12 months follow-up was 60%	
Merolla et al.	2017	1. PRP (unknown)	PRPS, BiomedDevice, Modena, Italy	Ultrasound Guided	50	At least 4 months	8, 24, 52, and 104 weeks	High risk	At week 104: VAS: 7.1 PRTEE: 69.2 Grip strength: 22.8	Both PRP injections and surgical treatments are effective in the short and medium term. However, PRP patients experienced a significant worsening of pain at 2 years whereas the surgical group enjoyed better long-term outcomes in terms of pain relief and grip strength recovery
		2. Arthroscopic release (surgical)	NR	NA	51	At least 4 months	8, 24, 52, and 104 weeks		At week 104: VAS: 2.1∗ PRTEE: 21.2∗ Grip strength: 48.4∗	
Thanasas et al.	2011	1. PRP (leukocyte enriched, type 1A)	Biometric GPS III	Ultrasound Guided	14	At least 3 months	6 weeks, 3 & 6 months	High risk	At 6 months: VAS score: 1.78(1.14–2.42) (At 6 weeks: VAS score: 2.35(1.83–2.87))∗ Liverpool elbow score: 9.32 (9.05–9.59)	The PRP group enjoyed faster pain relief than the AWB group.
		2.AWB		Ultrasound Guided	13	At least 3months	6 weeks, 3 & 6 months		At 6 months: VAS score: 2.53 (1.89–3.17) At 6 weeks: VAS score: 3.5(2.82–4.18))∗ Liverpool elbow score: 8.85 (8.40–9.30)	
Linnanmäki et al.	2020	1. PRP (unknown)	Centrifugation machine: Hettich Rotofix A32	Manual Palpation	31	At least 3 months	4, 8, 12, 26, and 52 weeks	High risk	At 1 year: VAS score: 2.7 ± 2.4 Dash score: 17.5 ± 18.2 Grip strength (kg): 7.6 ± 9.1	After 1 year post treatment, there was no improvement in terms of pain or function in both PRP and AWB group of patients compared with those who were given a saline injection.
		2.AWB		Manual Palpation	38	At least 3 months	4, 8, 12, 26, and 52 weeks		VAS score: 2.1 ± 2.1 Dash score: 24.1 ± 18.9 Grip strength(kg): 6.0 ± 10.3	
		3. Saline		Manual Palpation	32	At least 3 months	4, 8, 12, 26, and 52 weeks		VAS score: 3.0 ± 2.5 Dash score: 16.0 ± 15.3 Grip strength (kg): 6.1 ± 8.6	
Gupta et al.	2020	1. PRP (Unknown)	NR	Manual Palpation	40	At least 3 months	6 weeks, 3 & 12 months	High risk	At 6 weeks: Mean VAS: 13.8∗; Mean DASH: 53.3∗; Mean MEPS: 74.5∗; Mean GSS: 73.4∗ At 1 year:: VAS score: 2.5 ± 5.5∗; DASH: 31.65 ± 3.87∗; MEPS: 98.25 ± 4.67∗; GSS: 112.75 ± 31.52∗	Patients who were treated with steroid injections had good short-term results at 6 weeks however the PRP treatment group had superior results at 3 and 12 months.
		2. Corticosteroids (Triamcinolone in 2% xylocaine)	NR	Manual Palpation	40	At least 3months	6 weeks, 3 & 12 months		At 6 weeks: Mean VAS: 44.5∗; Mean DASH: 64.2∗; Mean MEPS: 88.0∗; Mean GSS: 89.3∗ At 1 year: VAS score: 13.5 ± 1.84∗; DASH: 40.1 ± 8.03∗; MEPS: 89.75 ± 12.62∗; GSS: 92.3 ± 24.68∗	
Martin et al.	2019	1. PRP (leukocyte poor)	NR	Ultrasound Guided	36	At least 3 months	6 & 12 months	High risk	At 1 year: % meeting VAS-P improvement (>25% reduction in score): 90.91% (Median VAS score = 2) % meeting DASH-E improvement(>25% reduction in score): 76% (Median DASH score: 9.17) Hypercholesteraemia and baseline vascularisation influenced outcomes.	The group of patients treated with PRP resulted in similar improvements to those receiving lidocaine.
		2. Lidocaine	NR	Ultrasound Guided	35	At least 3months	6 & 12 months		% meeting VAS-P improvement: 76.0% (Median VAS score = 2) % meeting DASH-E improvement: 70.83% (Median DASH score: 7.50) Hypercholesteraemia and baseline vascularisation influenced outcomes	
Watts et al.	2018	1. PRP (leukocyte rich)	Zimmer Biomet Recover Platelet Separation Kit and GPS III	Manual Palpation	40	At least 6 months	6,12,24 and 52 weeks	High risk	At 1 year: PRTEE pain score: 17/50 (Pre-test: 32/50)∗ PRTEE function score; 10/50 (Pre-test: 28/50) PRTEE total score: 26/100 (Pre-test:58/100) DASH score: 22/100 (Pre-test: 47/100)	Both PRP injections and surgical treatments are led to similar functional outcome. However, the surgically treated patients had lower pain scores at 12 months.
		2. Open surgical release		NA	41	At least 6 months	6,12,24 and 52 weeks		At 1 year: PRTEE pain score: 9/50 (Pre-test: 33/50)∗ PRTEE function score: 7/50 (Pre-test: 29/50) PRTEE total score: 16/100 (Pre-test: 62/100) DASH score: 12/100 (Pre-test: 45/100)	
Lim et al.	2017	1. PRP (unknown)	HUONS, Sungnam, Korea	Ultrasound Guided	55	At least 3 months	4 weeks, 3 & 6 monts	High risk	After 4 weeks: Change in VAS score: 40.6; Change in Mayo score: 8.42; Change in MRI grade: 1.11 At 24 weeks, VAS score, Mayo score and MRI grade improved significantly∗. TGF-beta levels increased from 3.92 to 112 ng/ml in the PRP. ∗ PDGF-AB, PDGF,BB levels also increased significantly TGF-beta level significantly correlated with Mayo clinic performance score and MRI grade improvement ∗	Compared to physiotherapy, the PRP group of patients reported improvements in pain and function. These improvements were consistent even after a follow up period of 6 months, without any complications.
		2. Physiotherapy	NR	NA	50	At least 3 months	4 weeks, 3&6 months		After 4 weeks: Change in VAS score: 40.6; Change in Mayo score: 8.42; Change in MRI grade: 1.11	
Varshney et al.	2017	1. PRP (Unknown)	Biomixer: Terumo Pempol D 601	Manual Palpation	33	Not recorded	1,2,6 and 12 months	High risk	After 6 months: VAS score: 0.69 ± 1.57 ∗(Preprocedure: 8.33 ± 1.08) Mayo score: 95.00 ± 9.39∗ (Preprocedure: 61.51 ± 6.75) No significant difference was found between the two groups at 1 and 2 months after the intervention	Compared to steroids, the PRP group of patients reported improvements in pain and function. These improvements were consistent even after a follow up period of 6 months, without any complications.
		2. Corticosteroid (methylprednisolone)		Manual Palpation	50	Not recorded	1,2,6 and 12 months		After 6 months: VAS score: 4.61 ± 1.46∗ (Preprocedure: 7.98 ± 1.16) Mayo score: 63.12 ± 6.40∗ (Preprocedure: 63.92 ± 7.32)	
Montalvan et al.	2015	1. PRP (Unknown)	Arthrex (Naples, FL, USA),	Ultrasound Guided	25	No more than 3 months	1,3,6 and 12 months	Low risk	After 12 months VAS score: 1.7 ± 1.5 (Change of −5.2 ± 1.3) Roles-Maudsley score: 2.3 ± 1.1 (Change of −1 ± 1.3) Patients with pain on ERCB contraction: 44% from 100% Patients with pain on EDC contraction: 32% from 88%	Compared to saline, PRP therapy led to no significant differences in pain relief.
		2. Saline solution	NR	Ultrasound Guided	25	No more than 3 months	1,3,6 and 12 months		After 12 months: VAS score: 1.8 ± 2.1 (Change of −5.4 ± 2.3) Roles-Maudsley score: 2.2 ± 0.9 (Change of −1.3 ± 0.9) Patients with pain on ERCB contraction: 52% from 100% Patients with pain on EDC contraction: 56% from 92%	
Behara et al.	2015	1. PRP (Leukocyte poor)	NR	Ultrasound Guided	15	At least 3 months	1,3,6 and 12 months	Unclear risk	At 6 months, the percentage of improvement for the VAS score was 67.3%, for the MMCPIE score was 40.6% and for the Nirschl score was 71.4%. ∗ At 1 year: VAS score, change from baseline: 83.2% MMCPIE, change from baseline: 47∗ Nirschl score, change from baseline: 76.6	Compared to Bupivacaine injections, the PRP group of patients reported improvements in pain and function. These improvements were consistent even after a follow up period of 6 months and 1 year.
		2. Bupivacaine injection	NR	Ultrasound Guided	9	At least 3 months	1,3,6 and 12 months		At 6 months, the percentage of improvement for the VAS score was 20.1%, for the MMCPIE score was 16.3% and for the Nirschl score was 31.1%. ∗ At 1 year: VAS score, % change from baseline: 45.6∗ MMCPIE, % change from baseline: 21.7∗ Nirschl score, % change from baseline: 56.3∗	
Gautam et al.	2015	1. PRP (Unknown)	NR	Manual Palpation	15	At least 6 months	2 weeks, 1,3 and 6 months	Unclear risk	At 6 months: VAS score: 1.6 ± 0.5∗ (Pre-injection: 7.1 ± 0.8)∗ DASH score: 32.0 ± 4.5∗ (Pre-injection: 69.7 ± 6.1) There were also significant differences between VAS and DASH scores for PRP from pre to post injection at 2 weeks and 6 months Oxford elbow score: 41.2 ± 2.7∗ (pre-injecton: 27.4 ± 3.9)∗ Modified Mayo score: 70.7 ± 3.0∗ (Pre-injection: 56.1 ± 6.9) Hand Grip Strength: 25.9 ± 6.2 (Pre-injection: 18.5 ± 5.1) Ultrasonography: 27% post-injection from 67% at pre-injection had a tear of the COE; 7% post-injection from 20% pre-injection had reduced thickness of the CEO tendon Modified Mayo score: 70.7 ± 3.0∗ (Pre-injection: 56.1 ± 6.9) Hand Grip Strength: 25.9 ± 6.2 (Pre-injection: 18.5 ± 5.1) Ultrasonography: 27% post-injection from 67% at pre-injection had a tear of the COE; 7% post-injection from 20% pre-injection had reduced thickness of the CEO tendon.	Compared to steroids, the PRP group of patients enable biological healing. However, steroids were found to provide short term pain relief but at the expense of increased tendon degeneration
		2. Corticosteroid (Methylprednisolone)	NR	Manual Palpation	15	At least 6 months	2 weeks, 1,3 and 6 months		At 6 months: VAS score: 2.9 ± 1.2∗ (Pre-injection: 7.0 ± 0.8) DASH score: 39.6 ± 1.0∗ (Pre-injection: 67.5 ± 6.9) There were also significant differences between VAS and DASH scores for PRP from pre to post injection at 2 weeks and 6 months Oxford elbow score: 36.3 ± 5.9∗ (pre-injecton: 31.2 ± 4.1)∗ Modified Mayo score: 61.5 ± 5.8∗ (Pre-injection: 56.8 ± 5.4) Hand Grip Strength: 23.3 ± 6.5 (Pre-injection: 19.2 ± 4.6) These scores peaked at 3 months and then deteriorated slightly at 6 months for 46.7% of the patients. Ultrasonography: 33% post-injection from 33% at pre-injection had a tear of the COE; 80% (12) post-injection from 13% (2) pre-injection had reduced thickness of the CEO tendon. Number of patients with cortical erosion at the lateral epicondyle increased from 9 to 11.	
Krogh et al.	2013	1. PRP (unknown)	Recover GPS II (Biomet biologics)	Ultrasound Guided	20	At least 3 months	1, 3, 6 and 12 months	Low risk	At 3 months: Change in PRTEE pain score from baseline: −6.0 ± 2.2 Change in PRTEE functional score from baseline: −16.6 ± 4.3 Change in colour doppler activity from baseline: −0.4 ± 0.2∗ Change in tendon thickness from baseline: 0.3 ± 0.1∗	Glucocorticoid injections were found to have an initial pain relieving effect at 1 month of follow up when compared to the other treatment groups. However, compared to saline injections, both the PRP and glucocorticoid group of patients reported no improvements in pain and function at the end of 3 months.
		2. Glucocorticoid (Triamcinoloon + lidocaine)		Ultrasound Guided	20	At least 3months	1,3, 6 and 12 months		At 3 months: Change in PRTEE pain score from baseline: −7.1 ± 2.2 Change in PRTEE functional score from baseline: −13.8 ± 4.3 Change in colour doppler activity from baseline: −3.0 ± 0.2∗ Change in tendon thickness from baseline: −0.2 ± 0.1∗ At 1 month: glucocorticoid reduced pain more than saline and PRP: Glucocorticoid vs saline: −8.1 (95% CI, −14.3 to −1.9); Glucocorticoid vs PRP: −9.3 (95% CI, −15.4 to −3.2)	
		3. Saline		Ultrasound Guided	20	At least 3 months	1,3, 6 and 12 months		At 3 months: Change in PRTEE pain score from baseline: −3.3 ± 2.2 Change in PRTEE functional score from baseline: −7.6 ± 4.3 Change in colour doppler activity from baseline: −1.0 ± 0.2∗ Change in tendon thickness from baseline: 0.6 ± 0.1∗	
Gosens et al.	2011	1. PRP (Leukocyte rich)	Recover system (Biomet biologics)	Manual Palpation	51	At least 6 months	4,8, 12, 26, 52 and 104 weeks	Low risk	At 2 years: VAS score: 21.3 ± 28.1∗ (Also significantly different at 4 weeks, 26 weeks and 1 year) DASH score: 17.6 +/24.0∗ (Also significantly different at baseline, 4 weeks and 1 year) PRP group was more often treated successfully∗ (defined as a reduction of 25% on the VAS score without a reintervention at 2 years)	Compared to steroids, the PRP group of patients reported improvements in pain and function. These improvements were consistent even after a follow up period of 2 years, without any complications.
		2. Corticosteroid		Manual Palpation	49	At least 6 months	4,8, 12, 26, 52 and 104 weeks		At 2 years: VAS score: 42.4 ± 26.8∗ (Also significantly different at 4 weeks, 26 weeks and 1 year) DASH score: 36.5 +/243.8∗ (Also significantly different at baseline, 4 weeks and 1 year)	
Creaney et al.	2011	1. PRP (Unknown)	Centrifuged with LC6; Sarstedt, Numbrecht, Germany	Ultrasound Guided	70	Not mentioned	1,3 and 6 months	Low risk	At 6 months: Mean improvement in PRTEE score: 35.8 (95% CI 30.3–41.4)∗(These improvements were greater than the predefined clinically significant improvement of 25) 66% success rate 10% rate of conversion to surgery	Compared to ABI, the PRP group of patients reported similar improvements in pain and function at 6 months of follow up.
		2. Autologous blood injection (ABI)	NR	Ultrasound Guided	60	Not mentioned	1,3 and 6 months		At 6 months: Mean improvement in PRTEE score: 46.8 (95% CI 42.1–51.5)∗(These improvements were greater than the predefined clinically significant improvement of 25) 72% success rate 20% rate of conversion to surgery	
Palacio et al.	2016	1. PRP (unknown)	NR	Manual Palpation	20	None mentioned	90 and 180 days	Unclear risk	∼81.7% of the patients who underwent treatment presented some improvement of symptoms. There was evidence that the cure rate was unrelated to the substance applied (p = 0.62). There was also intersection between the confidence intervals of each group, thus demonstrating that the proportions of patients whose symptoms improved were similar in all the groups.	After 90 and 180 days post treatment, there was no evidence of a superior form of treatment when assessed using the DASH and PRTEE questionnaires.
		2. Neocaine	NR	Manual Palpation	20	None mentioned	90 and 180 days			
		3. Dexamethasone	NR	Manual Palpation	20	None mentioned	90 and 180 days			
Schoffl et al.	2017	1.PRP (Leukocyte poor)	Arthrex ACP	Manual Palpation	18	At least 3 months	4 weeks and 6 months	High risk	At 4 weeks: DASH score: 40.2 ± 18.2 (Pre-therapy: 41.0 ± 18.0) At 6 months: DASH score: 30.1 ± 20.2	There was no evidence of a statistically significant difference between PRP and the placebo group.
		2. Saline		Manual Palpation	18	At least 3 months	4 weeks and 6 months		At 4 weeks: DASH score: 30.6 ± 18.8 (Pre-therapy: 36.4 ± 17.7)∗ (Decrease in DASH score was significantly greater in placebo compared to PRP group at 4 weeks) At 6 months: DASH score: 25.8 ± 22.6 (Mean decrease of 15.3∗)	
Yadav et al.	2015	1.PRP (Unknown)	9001:2000 ISO certified R-23 centrifuge	Manual Palpation	30	1–6 months	15 days, 1 month & 3 months	Unclear risk	At 3 months: Mean VAS score: 1.6∗ (Baseline: 7.6) Mean grip strength: 156.66∗ (Baseline: 74.66) Mean qDASH: 34.16∗ (Baseline: 88) All of these outcomes were also significantly improved from baseline at 15 days & 1 month as well. At 3 months, this improvement was significantly better than the corticosteroid group.	Both treatments were effective however at 3 months, PRP showed significantly better improvement suggesting longer duration efficacy.
		2. Methylprednisolone		Manual Palpation	30	1–6 months	15 days. 1 month & 3 months		At 3 months: Mean VAS score: 2.8∗ (Baseline: 7.7) Mean grip strength: 136.16∗ (Baseline: 74.5) Mean qDASH: 44.33∗ (Baseline: 88) All of these outcomes were also significantly improved from baseline at 15 days & 1 month as well.	
Omar et al.	2012	1.PRP (Unknown)	JMS hemoscale and Forma Scientific, Marietta, oH.	Manual Palpation	15	Not mentioned	6 weeks	Unclear risk	At 6 weeks: VAS score: 3.8 ± 1.9∗ (Baseline: 8.0 ± 1.4) DASH score: 19.9 ± 12.9 ∗(Baseline: 58.9 ± 10.5)	Both PRP & steroids showed statistically significant improvements in outcomes at 6 weeks. However, there was no statistically significant difference between the 2 groups
		2. Corticosteroid		Manual Palpation	15	Not mentioned	6 weeks		At 6 weeks: VAS score: 4.3 ± 2.1∗ (Baseline: 8.6 ± 1.6) DASH score: 20.2 ± 14.0∗ (Baseline: 57.3 ± 10.3)	

*PRP*, platelet-rich plasma; *VAS*, visual analog scale; *PRTEE*, Patient-Rated Tennis Elbow Evaluation; *DASH*, Disabilities of the Arm, Shoulder and Hand; *PPT*, pressure pain threshold; MEPS, Mayo Elbow Performance Score. *GSS,* Grip strength score; *ECRB,* Extensor Carpi Radialis Brevis; *EDC,* Extensor digitorum communis; *MMCPIE,* modified Mayo clinic performance index for elbow; NR, Not Recorded; NA, Not Applicable.

∗ = Significant at P < 0.05.

∗∗ = Assessed using the modified Cochran's Collaboration Risk of Bias Assessment Tool for RCTs.

**Fig. 1:**
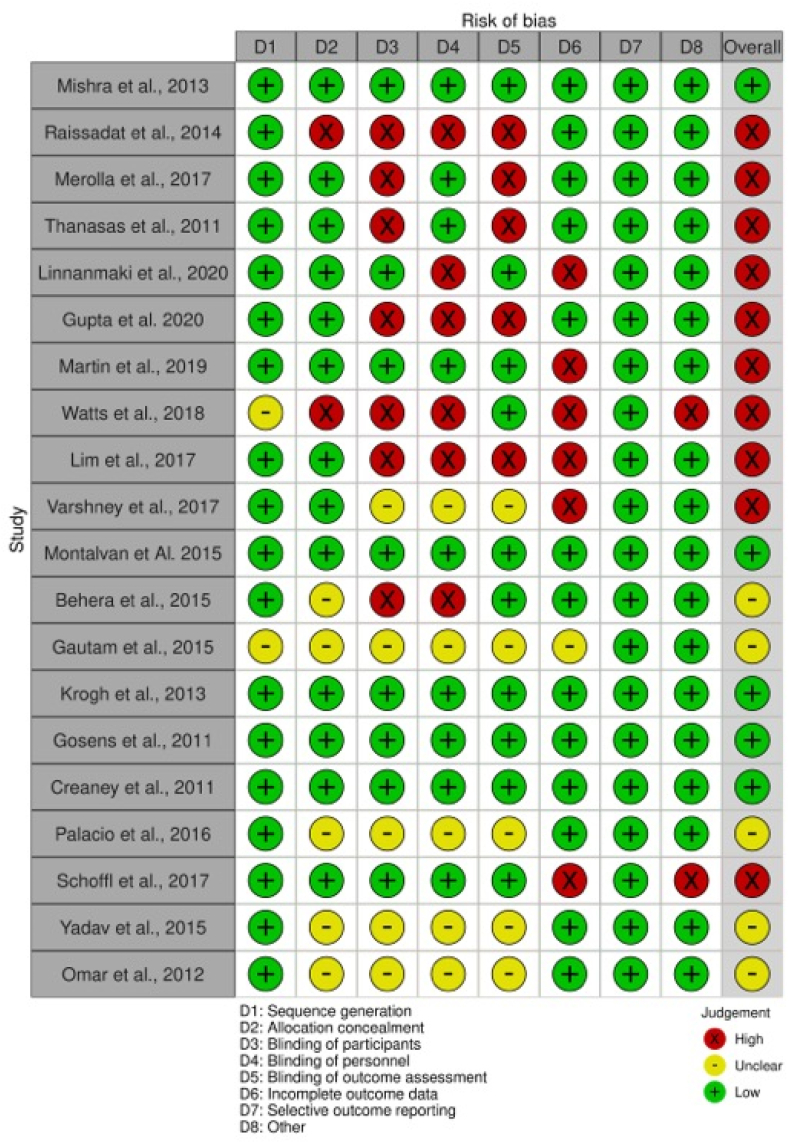
Summary of the included RCTs' risk of bias.

The quality of the RCTs included were analysed for their study design. The risk of bias for each RCT was evaluated using the modified Cochrane Collaboration Risk of Bias Assessment Tool for randomised trials (adapted from Higgins and Altman).^14^ 2 reviewers individually assigned each RCT to have a classification of either “high risk”, “low risk”, or “unclear risk” in various aspects of each study as summarised in Fig. 1. Further discussion with the senior reviewers were done to resolve any discordance between the 2 initial reviewers. A RCT was classified with an overall low bias risk if the criteria for “low risk” was met for the following aspects of the study: participant blinding (D3), selection bias (D1 and D2), attrition bias (D6), and reporting bias (D7).^15^

## 3 Results

**Fig. 2:**
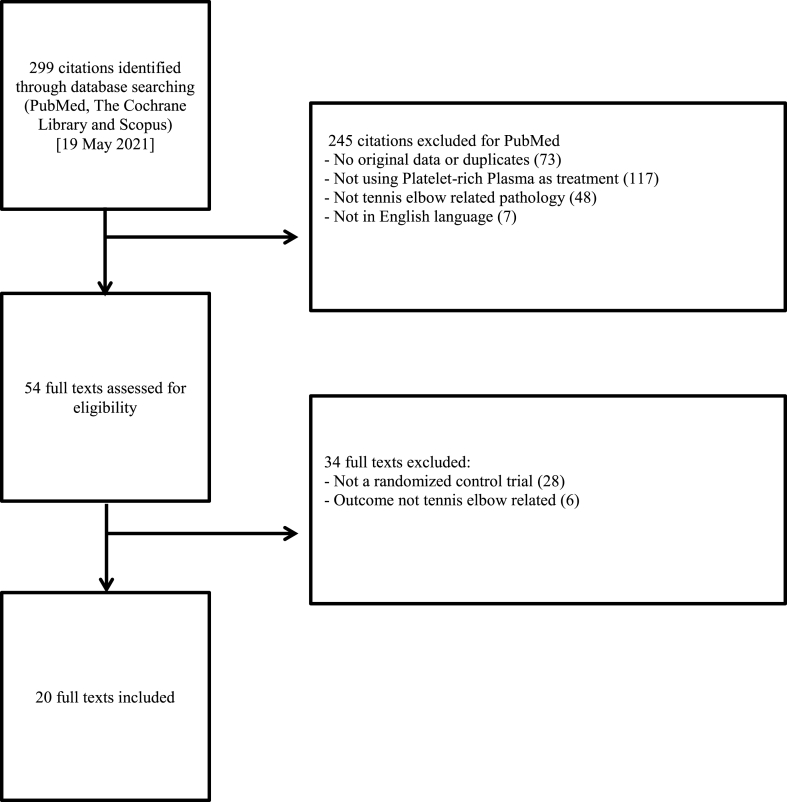
PRISMA Flow diagram of the review and selection process of publications.

The PubMed, Scopus and Cochrane database searches yielded 299 manuscripts collectively. These manuscripts underwent a two-stage selection process by two independent reviewers (Fig. 2). In the first step, all 299 abstracts were screened against the criterions for inclusion and exclusion. Abstracts without original data, not utilising PRP as therapy for TE, not in English language and duplicates were excluded. Secondly, the manuscripts were retrieved in full and subsequently analysed for eligibility. Studies not randomised in a control trial or failed to record comparison outcomes of PRP and the control group were excluded. Bibliographies of relevant published papers were also reviewed with the same two-stage process (Fig. 2) to detect other relevant studies not captured by the primary search.

### 3.1 Overview of studies

In this review, a total of 20 RCTs were included with a total of 1520 TE patients analysed and followed up for a minimum of 6 weeks (range 6–104 weeks).^16^, ^17^, ^18^, ^19^, ^20^, ^21^, ^22^, ^23^, ^24^, ^25^, ^26^, ^27^, ^28^, ^29^, ^30^, ^31^, ^32^, ^33^, ^34^, ^35^ The average number of patients recruited for each RCT was 76 (range 24–230 patients) with the largest RCT conducted being a multicentre study including 230 patients by Mishra et al.^16^

The RCTs included in this review compared PRP with various treatment strategies for TE ranging from placebo saline injections to conservative physiotherapy to surgical release. In the patients who underwent PRP injection to the ECRB tendon or tendon sheath, 8 studies (40%) administered PRP under ultrasound guidance while the remaining 12 studies (60%) performed manual palpation. The most common comparison group within these RCTs was corticosteroid injections. Only 3 RCTs compared PRP with multiple treatments modalities within the same study.

With regards to preparation, PRP can be formulated to be either leukocyte enriched or deficient. Of the 20 RCTs, only 8 studies documented the formulation of the PRP therapy used. 5 RCTs utilised leukocyte-rich PRP whereas the other 3 RCTs used leukocyte-poor PRP. Not one study compared the different formulations of PRP as separate treatment groups.

The included studies recruited TE patients with varying duration of symptoms. 14 RCTs exclusively involved TE patients who were chronically symptomatic for longer than 3 months. 4 RCTs did not document the duration of symptoms of their patients.

All RCTs recorded the length of follow up post treatment. 16 RCTs followed up before 4 weeks and had multiple subsequent follow ups. 2 RCTs followed up significantly later at 90 days and 6 months respectively. 2 RCTs followed up just once after 6 weeks and 2 years.

In terms of blinding, only 1 RCT completely blinded all 3 parties involved with the study i.e. patient, assessor and radiologist. 9 RCTs arranged for single blinding while 6 RCTs performed double blinding. 4 RCTs failed to document any form of blinding.

### 3.2 Bias risk assessment of studies

All 20 RCTs included in this review underwent a thorough risk of bias assessment of which a summary of the analysis is reflected in Fig. 1. Across the RCTs assessed, 5 studies (25%) were classified as low risk of bias, 5 studies (25%) were unclear, whilst the remaining 10 studies (50%) had a high risk of bias.

### 3.3 PRP vs steroid therapy

A total of 8 RCTs compared the use of PRP with steroids as therapy for TE.^17^, ^18^, ^19^, ^20^, ^21^, ^22^, ^23^, ^24^ 4 RCTs found PRP to be more effective than corticosteroid injection of which 1 RCT had a low risk of bias.^17^^,^^19^^,^^20^^,^^23^ Only 1 of the 4 RCTs reporting similar efficacy between PRP and steroids had a low risk of bias.^18^

### 3.4 PRP vs autologous whole blood (AWB) therapy

A total of 4 RCTs have been performed to compare PRP with AWB therapy.^25^, ^26^, ^27^, ^28^ The only low risk of bias RCT reported PRP to be similarly efficacious as AWB at treating TE.^25^ The only RCT suggesting both PRP and AWB had no therapeutic effect was at high risk of bias.^28^

### 3.5 PRP vs surgical management

In summary, only 2 RCTs were found to have compared PRP with surgical interventions. Patients in these studies found surgical management to be superior in terms of pain relief compared to PRP, however both studies had a high risk of bias.^29^^,^^30^

### 3.6 PRP vs physiotherapy

Only 1 RCT was found to have compared PRP with physiotherapy which showed superior pain and functional results in the PRP group however, this study had a high bias risk.^31^

### 3.7 PRP vs anaesthesia

A total of 3 RCTs were found to have compared PRP with anaesthesia however all 3 studies had either a high or unclear risk of bias.^22^^,^^32^^,^^33^ The only RCT reporting PRP to be superior in terms of pain relief and functional scores had an unclear risk of bias.^32^

### 3.8 PRP vs placebo

In summary, 5 RCTs were found to have compared PRP with various types of placebo including percutaneous needle tenotomy and saline injections. 4 RCTs compared PRP with saline injections however all 4 studies reported no difference in pain or functional scores.^18^^,^^28^^,^^34^^,^^35^ The only RCT that compared PRP with dry needling as an active control group reported superior effects with PRP.^16^ This RCT had a low risk of bias.

## 4 Discussion

Overall, the primary finding of the current study demonstrate that PRP when compared to a variety of other treatment modalities, including placebo, physiotherapy, injections containing corticosteroid, PRP and AWB as well surgical interventions fail to demonstrate a significant improvement in pain or functional outcomes in the management of TE.

The most common treatment group that PRP was compared with was corticosteroid injections. Gosens et al. reported superior relief of pain and restoration of function more effectively with PRP than steroid injections in patients with chronic TE.^17^ This study had a low bias risk. Yadav et al. reported both PRP and steroids to be effective at pain relief and improving functional outcomes but the PRP group enjoyed significantly better improvements at the 3 months follow up period.^23^ This study has an unclear risk of bias. Conversely, Krogh et al. reported that while PRP and steroid therapy were similar to saline in terms of alleviating pain at 3 months follow up, steroid therapy provided superior pain relief in comparison to the other treatment groups at 1 month follow up.^18^ This study had a low bias risk. Gupta et al. reported greater short term pain management and performance scores at 6 weeks with steroid injections, but patients receiving PRP injections enjoyed superior results in the longer term.^19^ However, 2 studies, Varshney et al. and Omar et al. found no difference between the PRP and steroid groups during the follow up period.^20^^,^^24^ Both of these studies had a high risk of bias. Gautam et al. reported that both PRP and steroids helped with significant improvement in pain and functional scores.^21^ However, PRP enabled biological healing for patients with chronic symptomatic TE, whereas patients treated with corticosteroids are at increased risk of tendon degeneration. There was an unclear level of bias risk with this study. Palacio et al. reported no statistical difference between PRP, steroid and anaesthetic therapy.^22^ This study had an unclear risk of bias.

In the past, steroid injections were popular and even considered to be the gold standard treatment for TE. However, recent studies have recommended against steroid therapy due to the short-lived efficacy as well as potential detrimental effects including high recurrence rates, dermal depigmentation and subcutaneous atrophy.^36^, ^37^, ^38^ Physiologically, steroids have been found to inhibit tenocyte proliferation and progenitor cell recruitment leading to reduced collagen synthesis and greater fatty tissue changes.^39^ It is therefore prudent to interpret studies that compare the efficacy of PRP and steroid therapy with caution. Instead, further studies should consider using anaesthetic injections as a more appropriate comparison treatment group to mitigate the steroids interference with healing mechanisms in TE patients.

Both AWB and PRP injections contain platelets which possess strong growth factors and granules vital to the healing process of chronic injuries. Theoretically, PRP contains a higher concentration of platelets than in AWB hence a superior effect in the repair process of tendinopathies.^26^ However, our findings show PRP to be similarly efficacious to AWB in treating TE. Creaney et al. reported a prospective single blinded RCT and found that both PRP and AWB provided comparable efficacy with a significant reduction in pain scores at 6 months.^25^ This study had a low bias risk. Raeissadat et al. performed a single blinded RCT and found that both PRP and AWB injection therapies were similarly efficacious with comparable pain and functional scores at every follow up interval.^26^ This study had a high bias risk. Thanasas et al. reported that PRP therapy was superior at pain reduction in TE patients compared to AWB in the short term at 6 weeks.^27^ However, in the longer term at 3 and 6 months, both AWB and PRP were statistically similar. This study had a high risk of bias. However, most recently in 2020, Linnanmäki et al. documented a RCT comparing PRP, AWB as well as placebo with saline as therapy for TE patients.^28^ Both PRP and AWB therapies did not help with pain or function in comparison to the saline group. This study had a high bias risk.

Surgical release or debridement of the damaged extensor tendon is typically considered after a failed trial of conservative treatment. Previous studies^40^ have documented high success rates of up to 97.7% however, only 2 RCTs were found to have directly compared surgical intervention with PRP. Our findings show that patients found surgery to be superior in terms of pain relief compared to PRP, the RCTs included were at high risk of bias especially because it was technically impossible to blind both patients and researchers. Merolla et al. compared the effects of PRP with arthroscopic debridement.^29^ They found that both were effective at improving both pain and performance initially. Conversely, surgical debridement was superior in the long-term. Watts et al. compared PRP with surgical release for refractory TE.^30^ They reported no statistical difference functionally however, surgical patients reported superior pain scores compared to the PRP group. Both studies had a high risk of bias.

Physiotherapy for the treatment of tennis elbow typically includes eccentric strengthening exercises for the wrist extensors and static stretching of the ECRB.^41^ While only up to 10% of TE patients eventually opt for surgical management,^42^ majority if not all patients would be offered a course of physiotherapy. However, only 1 RCTs compared PRP with physiotherapy and reported PRP led to superior pain and functional scores in TE patients.^31^ These improvements were reported to be sustained longer than 6 months of follow up, without complications in any patient.

Needle tenotomy can be performed as a standalone procedure (“dry needling”) or part of a combined intervention such as with local anaesthetic agents. Theoretically, tenotomy in itself may induce healing via microtrauma.^32^ However, multiple studies have performed needle tenotomy as a control or placebo group to compare against PRP. Palacio et al. compared PRP, steroid and anaesthetic therapy but reported no evidence of any statistical difference between these treatment modalities.^22^ This study had an unclear risk of bias. Martin et al. compared PRP therapy with lidocaine as tenotomy adjuvants in recalcitrant TE patients.^32^ They reported no evidence of statistical differences in terms of pain and functional scores. This study had a high risk of bias. Behera et al. compared PRP therapy with bupivacaine.^33^ They reported superior results with PRP injections in terms of pain relief and functional scores. This study had an unclear risk of bias. Linnanmäki et al. documented that PRP and other treatment modalities were similar to saline in terms of alleviating pain and function in TE patients.^28^ This study had a high bias risk. 3 studies examined the effects of PRP as compared to saline injections and reported no statistical difference in both pain and functional outcomes.^18^^,^^34^^,^^35^ 2 studies^18^^,^^34^ had a low risk of bias while 1 had a high risk of bias.^35^ Mishra et al. compared the effects of PRP with simple tendon needling alone.^16^ They reported a greater efficacy with PRP compared to dry needling alone as treatment for TE. This study had a low bias risk.

Analysing the methodology and quality of the 20 RCTs, only 5 studies (25%) met the criteria to be classified having a low bias risk. The bias risk in the remaining RCTs severely limits the interpretability of the results obtained. Notably, there was high risk of bias with regards to the blinding of participants, personnel including clinicians and researchers, outcome of assessment in almost half of the RCTs. Whilst this can be partially attributed to the nature of treatment groups i.e. surgical intervention compared to PRP injections, multiple studies were performed with ambiguous blinding methodology. For instance, multiple studies recorded single,^26^ or triple blinding,^22^ but failed to document the blinding procedures in the manuscript. 4 other studies failed to blind at all.^21^^,^^23^^,^^24^^,^^33^ Consequently, inconsistent blinding amongst these RCTs led to the classification of high risk for bias.

The vast spectrum of treatment options available demonstrates that TE encompasses a range of heterogeneous conditions which affect the common extensor tendons with varying severity. Physiologically, tendons possess the capability to stretch in response to increasing forces. However, a microtear will ensue if this force exceeds the tendon's elastic threshold. Repetitive stress and overuse may result in multiple microtears leading to degenerative changes also known as tendinosis. Eventually, a full-thickness tendon tear may ensue with gradual degradation of the tendon. In general, there are 4 distinct grades of tendinopathy as succinctly summarised by Bhabra et al.^6^ Using basic science principles, they proposed a treatment algorithm to help clinicians manage TE appropriately depending on severity of tendinopathy.^6^ Briefly, grade 1 tendinopathy only requires rest and activity modification to avoid further damage. Grade 2 tendinopathy is classified according to the presence of immature vascular hyperplasia hence indicating PRP or AWB injections. In grade 3 tendinopathy, the loss of cells due to apoptosis and autophagy indicates replacing with autologous cell therapy. Finally, in grade 4 tendinopathy, surgical repair may be required to mechanically restore the collagen matrix. Consequently, the efficacy of PRP in TE will depend on the grade of tendinopathy as well as healing stage. Given this broad spectrum of severity in TE patients, RCTs should aim to identify and specify the grade of tendinopathy in their patient cohort to avoid bias in the interpretation of results. However, multiple studies failed to document the chronicity of symptoms or if any prior intervention was trialled in their TE patients let alone the grade of tendinopathy. This heterogeneity of patients in the RCTs makes their findings additionally difficult to generalise.

The results of this review must be considered in light of some limitations. Firstly, the heterogeneity of patients and the severity of their condition was not accounted for. Furthermore, only 7 studies documented the preparation and formulation of the PRP therapy used. The 2 main types of PRP include leukocyte-rich and leukocyte-poor PRP of which the former consists of a high number of leukocytes and is pro-inflammatory, whilst the latter is devoid of neutrophils making it anti-inflammatory in nature.^43^ Given that proteases secreted by leukocytes can affect growth factors secreted by platelets, superior results might be achieved using pure PRP instead.^44^ This was supported in a recent review which also suggested leukocytes in PRP may affect the efficacy provided by PRP.^45^ It is therefore imperative that the formulation of PRP therapy used is documented so that further research will be able to accurately compare such studies. It is however unfortunate that not one RCT, in this review, was found to compare the different formulations of PRP in separate treatment groups. Additionally, it should also be noted that only 7 studies documented the brand and type of machine that was used to prepare PRP therapy. This apparent lack of homogeneity of PRP preparations could in-part justify the reported differences in outcomes amongst the RCTs in this review.

Despite the heterogeneity of RCTs included in this review, several high level evidence studies including systematic reviews and meta-analysis have been previously published to analyse the efficacy of PRP in TE patients. In 2017 and 2018, multiple studies found that while steroid therapy may be associated with greater initial benefit, PRP therapy have superior long term outcomes.^46^^,^^47^ Interestingly, a recent systematic review published in 2021 concluded that PRP therapy provided similar improvements to surgical interventions in terms of functional scores and pain relief for TE patients in the initial phase.^48^ These abovementioned studies demonstrate a high level of evidence suggesting therapeutic efficacy of PRP therapy in TE patients. However, the literature is riddled with an abundance of mixed and contrasting evidence as well. A recent meta-analysis reported insufficient evidence to recommend the usage of PRP for TE.^49^ Another meta-analysis found PRP to be only minimally effective when compared to other forms of minimally invasive therapies for TE such as steroids, AWB injections, local anaesthetics, dry needle and even saline injections.^50^ The insufficient evidence of PRP therapy coupled with the relatively high costs of PRP and its accompanying preparational equipment makes it difficult to justify PRP therapy for TE currently.

## 5 Conclusion

Overall, our primary finding is that there is limited evidence to recommend the use of PRP therapy in terms of pain relief and function for TE. Further research with larger cohorts and longer duration of follow up are required to establish the optimal formulation and administration of PRP injections. Additionally, proper documentation of TE patients and the chronicity of their symptoms, pain and functional evaluation scores need to be standardised before concrete recommendations can be made regarding the therapeutic efficacy of PRP.

## Ethical approval

No Institutional Review Board approval was required for this study.

## Funding

This research did not receive any specific grant from funding agencies in the public, commercial, or not-for-profit sectors.

## Credit author statement

**Joshua Wong Rui Yen:** Visualisation, Formal analysis, Investigation, Data curation, Writing – original draft preparation, Writing- review and editing, Project administration. **Esme Toth:** Formal analysis, Investigation, Data curation, Writing – original draft preparation, Writing- review and editing. **Kannan Rajesparan:** Formal analysis, Methodology, Resources, Writing – review and editing, Supervision. **Abbas Rashid:** Visualisation, Formal analysis, Conceptualization, Methodology, Resources, Writing – review and editing, Supervision, Project administration

## Declarations of competing interests

None.
